# Exosomal transfer of proteins and RNAs at synapses in the nervous system

**DOI:** 10.1186/1745-6150-2-35

**Published:** 2007-11-30

**Authors:** Neil R Smalheiser

**Affiliations:** 1 University of Illinois-Chicago, UIC Psychiatric Institute MC912, 1601 W. Taylor Street, Chicago, IL 60612, USA

## Abstract

**Background:**

Many cell types have been reported to secrete small vesicles called exosomes, that are derived from multivesicular bodies and that can also form from endocytic-like lipid raft domains of the plasma membrane. Secretory exosomes contain a characteristic composition of proteins, and a recent report indicates that mast cell exosomes harbor a variety of mRNAs and microRNAs as well. Exosomes express cell recognition molecules on their surface that facilitate their selective targeting and uptake into recipient cells.

**Results:**

In this review, I suggest that exosomal secretion of proteins and RNAs may be a fundamental mode of communication within the nervous system, supplementing the known mechanisms of anterograde and retrograde signaling across synapses. In one specific scenario, exosomes are proposed to bud from the lipid raft region of the postsynaptic membrane adjacent to the postsynaptic density, in a manner that is stimulated by stimuli that elicit long-term potentiation. The exosomes would then transfer newly synthesized synaptic proteins (such as CAM kinase II alpha) and synaptic RNAs to the presynaptic terminal, where they would contribute to synaptic plasticity.

**Conclusion:**

The model is consistent with the known cellular and molecular features of synaptic neurobiology and makes a number of predictions that can be tested in vitro and in vivo.

**Open peer review:**

Reviewed by Etienne Joly, Gaspar Jekely, Juergen Brosius and Eugene Koonin. For the full reviews, please go to the Reviewers' comments section.

## Background

The purpose of this paper is discuss the potential role of the endosome-derived vesicle known as the secretory exosome as a means of intercellular signaling of proteins and RNAs in the nervous system. The exosome is a relatively well-characterized entity that has discrete mechanisms of formation and secretion, is secreted by diverse cell types, is regulated by physiological conditions, and has been implicated in intercellular transfer of specific signaling proteins. Recent studies have reported that secretory exosomes (and microvesicles shed by cells) contain a subset of cellular mRNAs and microRNAs as well, which can be transferred to and translated within recipient cells.

### 1. Secretory exosomes as a major pathway of cell-cell communication

**Figure 1:**
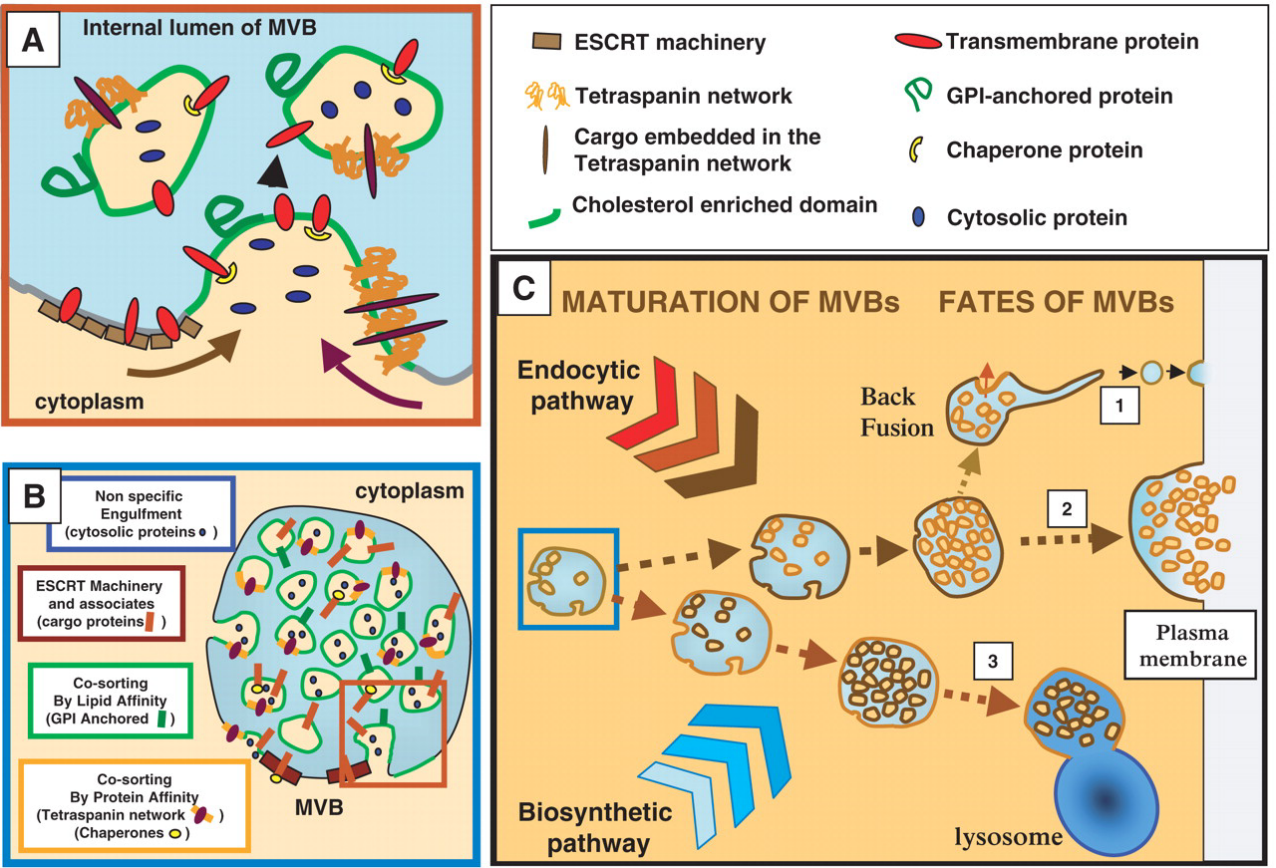
**Biogenesis of exosomes**. A: At the limiting membrane of MVBs, several mechanism act jointly to allow specific sorting of transmembrane, chaperones, membrane associated and cytosolic proteins on the forming ILVs. B: Presence of several sorting mechanisms may induce heterogeneity in the population of ILVs in single MVBs by acting separately on different domains of the limiting membrane. C: Receiving lipids and proteins from the endocytic and the biosynthetic pathway, different subpopulations of MVBs may be generated whose composition confers them different fate: (1) back fusion of the ILVs with the limiting membrane. During this process molecules previously sequestered on the ILVs are recycled to the limiting membrane and to the cytosol. Change in the composition of the limiting membrane may be responsible for the tubulation allowing plasma membrane expression of endosomal proteins. (2) Unknown mechanism may lead MVBs toward the plasma membrane where proteins such as SNAREs and synaptogamins would allow their fusion and the consequent release of the ILVs in the extracellular medium as exosomes. (3) Similarly, the composition of the limiting membrane would preferentially induce fusion of MVBs with lysosomes leading to the degradation of the molecules sorted on ILVs. Reproduced from ref. 1 with the permission of Oxford University Press.

First, some terminology: Secretory exosomes are vesicles formed via a specific intracellular pathway involving multivesicular bodies or endosomal-related regions of plasma membrane [1,2] (see fig. 1). They have a discrete size (generally 30–90 nm in different cell types), characteristic buoyant density (~1.1–1.2 g/ml), and lipid composition (similar to membrane lipid rafts, they are rich in cholesterol, sphingomyelin and ganglioside GM3, which confers resistance to Triton detergent and sensitivity to saponin). Exosomes express certain marker proteins (e.g., Alix and Tsg101, which are involved in endosomal-lysosomal sorting) but lack markers of lysosomes, mitochondria or caveolae. To summarize the classic pathway that forms exosomes, van Niel et al. said:

"Multivesicular bodies (MVBs), and their intraluminal vesicles (ILVs), are involved in the sequestration of proteins destined for degradation in lysosomes. An alternative fate of MVBs is their exocytic fusion with the plasma membrane leading to the release of the 50–90 nm ILVs into the extracellular milieu. The secreted ILVs are then called exosomes." [1].

Exosomes also express specific cell-surface proteins including integrins and cell adhesion molecules, so they have the means to bind selectively to, and be taken up by, specific recipient cell types [1-3].

As such, secretory exosomes can be distinguished from cell debris released from dead or dying cells, and from microvesicles, which are larger (up to 1 micron), denser, and occur via pinching-off from the plasma membrane; microvesicles may contain mitochondria, lysosomes and even DNA. Note that some authors use the term "microvesicle" generically to include secretory exosomes and even miscellaneous intracellular vesicles resembling synaptic vesicles. Conversely, the term "exosome" is also used by molecular biologists in an entirely unrelated context, to refer to intracellular RNA degradation complexes. Throughout this review, the term "exosome" will refer to secretory exosomes.

Recently, Valadi et al. [3] reported that secretory exosomes released from mast cells *in vitro *contain not only a distinctive set of proteins, but a population of mRNAs and microRNAs as well. The RNAs appeared to be compartmentalized insofar as their relative abundance within exosomes differed significantly from the overall profile of RNAs expressed within the cells [3]. Indeed, certain RNAs were highly enriched within the exosomes, suggesting that they were sorted into this compartment specifically. They also claimed that "after transfer of mouse exosomal RNA to human mast cells, new mouse proteins were found in the recipient cells, indicating that transferred exosomal mRNA can be translated after entering another cell." [3].

Secretory exosomes are attractive vehicles for intercellular RNA transfer, since they should provide a protected environment ensuring their stability despite the presence of extracellular RNAses. However, the Valadi et al. paper [3] is not free of potential criticism. For example, although most of the proteins described within their exosomes were consistent with other reports [4-10], their exosomal preps were atypical in the sense that they contained many ribosomal proteins. This is all the more puzzling since they did not detect ribosomal RNA 18S and 28S bands within their prep by gel electrophoresis (though possibly this might have been caused by partial RNA degradation). Although they did show that RNAs co-localized with low density fractions characteristic of exosomes (1.1–1.2 g/ml), their prep did not employ density gradients as part of the initial purification, but also contained material which ran at higher densities, thus a portion of the RNAs might have derived from a mixed population of microvesicles or other particles.

It will be necessary to confirm and extend the findings of Valadi et al. before they can be universally accepted. However, their report is consistent with observations that specialized endocytic pathways are involved in intracellular movement of RNAs during systemic RNA silencing in *C. elegans *and *Drosophila *[11-15]. As well, other investigators have independently reported that microvesicles shed by various cell types can also mediate mRNA transfer. For example, Ratajczak et al. examined microvesicles that were "enriched in exosomes"; the transferred mRNA was translated within recipient cells [16,17]. Baj-Krzyworzeka et al. examined microvesicles shed by tumor cells, which apparently were not enriched in exosomes; these contained both proteins and mRNA, and were internalized within and had survival-promoting effects on recipient cells [18]. Deregibus et al. isolated microvesicles from endothelial precursor cells and showed that they had effects (promoting cell survival and formation of capillary-like structures) in vitro that were destroyed by incubation with RNAse. The microvesicles contained mRNA, and the mRNA transfer was verified using GFP-tagged mRNA [19]. Finally, Tran et al. [20] induced expression of long complementary RNAs with the potential to form dsRNA in cultured cells, and found that the observed gene silencing effect was transferable via conditioned medium to recipient control cells. However, they did not identify the RNA species responsible for this effect, nor the mechanism by which cells would have released the RNAs.

Although secretory exosomes are the focus of this review, it is worth pointing out that other cellular mechanisms may also potentially facilitate intercellular RNA signaling in mammalian systems. For example, tunneling nanotubes can provide cytoplasmic bridges between cells, at least in vitro [21]. The *sid-1 *gene product discovered in *C. elegans *is a dsRNA-permeable pore that is necessary for systemic silencing of RNAs in invertebrates [13,22] and its mammalian homologue has been demonstrated to be functional in mammalian cells in vitro [23,24]. Some gap junctions can allow small oligonucleotides to pass through, including siRNAs [25,26]. Finally, it has been proposed that RNAs may be secreted into extracellular spaces despite their susceptibility to degradation by extracellular RNAses [27]; indeed, extracellular RNAses appear to have a regulatory role in cell physiology, which may be a clue that the secreted RNAs comprise a form of cell-cell communication [27]. This is also consistent with the observation that Toll-like receptor 3 expressed on cell surfaces is bound and activated by a variety of RNAs, including dsRNAs, mRNAs and siRNAs [28]. A secreted viral messenger protein, VP22, produced during HSV infection of mammalian cells, binds mRNAs and transfers them to recipient cells where they are translated [29].

### 2. Biological features of exosomes

The earliest role proposed for secretory exosomes was to shed unwanted proteins from cells undergoing terminal differentiation [30]. Although this perspective may apply in certain situations within the nervous system (see below), the protein composition of exosomes does not resemble a garbage dump, but rather is more consistent with a positive role in communication with other cells [4-10]. Exosomes express specific integrins, tetraspanins, MHC Class I and/or Class II antigens, CD antigens and cell-adhesion molecules on their surfaces, which may facilitate their uptake by specific cell types. Exosomes contain a variety of cytoskeletal proteins, GTPases, clathrin, chaperones, and metabolic enzymes (but mitochondrial, lysosomal and ER proteins are excluded, so the overall profile does not resemble the cytoplasm). They also contain mRNA splicing and translation factors. Finally, exosomes generally contain several proteins such as HSP70, HSP90, and annexins that are known to play signaling roles yet are not secreted by classical (ER-Golgi) mechanisms.

Exosomes can arise not only from fusion of multivesicular bodies with the plasma membrane (a "delayed mode") but also directly by budding from endocytic-like cholesterol-rich domains (lipid rafts) of the plasma membrane (an "immediate mode") [31,32]. Note that membrane lipid rafts are not synonymous with caveolae since there are numerous examples of plasma membrane domains [33] as well as endocytic vesicles that are cholesterol-rich, light in density and yet lack caveolin and other caveolar markers. However, it is not clear that all light endocytic vesicles fall into one family: for example, "enlargeosomes", intracellular cholesterol-rich secretory vesicles that expand the surface area of certain cell types, can be labeled with markers taken up by endocytosis but are not obviously related to the endosome-lysosome pathway, nor does it appear that they derive from MVBs [34].

Sorting of proteins into exosomes appears to be selective, albeit no single set of rules applies [35,36]. Mono-ubiquitination appears to be a tag for certain transmembrane proteins to be sorted into the endosomal-lysosomal pathway via ESCRT I, II and III proteins [35,36]. Furthermore, exosomes contain cholesterol-rich membrane rafts, with which some proteins are selectively associated. Recently, Fang et al. proposed that proteins which exhibit higher-order oligomerization (i.e. oligomerization of oligomers) and which also associate with the plasma membrane, are preferentially sorted into exosomes [32].

Proteins that contain signal peptides generally are secreted via the ER-Golgi pathway, and few such proteins have been detected within exosomes. In contrast, there appears to be an interesting relationship between exosomes and the heterogeneous class of proteins that lack signal peptides. These so-called nonclassically secreted proteins have been proposed to be secreted via one or more of the following 5 pathways [37,38]:

1. secretory lysosomes, associated with late endosomal-lysosomal markers, inhibited by chloroquine;

2. secretory exosomes;

3. plasma membrane microvesicles;

4. plasma membrane transporters (pumps or pores);

5. direct translocation through membranes of the so-called messenger proteins [38], including tat, VP22 and other viral proteins, as well as numerous homeodomain proteins, all of which contain short highly arginine-rich domains that engage the lipid portion of the plasma membrane [39].

Interestingly, the same or closely related proteins may utilize more than one of these pathways under different situations. For example, chick CNTF appears to be secreted via endocytic vesicles that are similar, if not identical, to secretory exosomes, though rat CNTF is not (because it lacks an internal hydrophobic domain present in chick) [40]. Whereas HSP70 is secreted via a lysosomal pathway in some cases [41], it is secreted via exosomes in others [42-44]. There has been a prominent controversy regarding how tat and other messenger proteins translocate through membranes – for example, depending on the nature of its linked cargo, the same protein may be taken up by a cell either via direct translocation or via endocytosis [45,46]. In the current context, it is worth noting that a wide variety of nonclassical proteins such as bFGF [47], the messenger protein Engrailed-2 [48], and galectins 1 and 3, which have been reported to be secreted by direct translocation across membranes, also appear to be secreted via intracellular cholesterol-rich vesicles or identified exosomes under certain conditions. As well, although ectodomain shedding of cell adhesion molecules is generally thought to occur via proteolytic cleavage events localized at the cell surface, in some cases the cleavage appears to occur within exosomes [49].

Release of exosomes is a regulated process. For example, exosomal secretion can be enhanced by stress conditions that elicit a p53 response [50]. In several cell types, including cultured cortical neurons [51], exosome secretion is stimulated by stimuli that raise intracellular calcium. Secretion of exosome-like microvesicles from adipocytes is regulated by hormones, redox, and nutrients [52]. The content of some individual proteins can be regulated independently as well; for example, IFN gamma induces the expression of HSP70 which is secreted via exosomes, but does not affect the rate at which exosomes are secreted per se [44].

### 3. Physiologic and pathologic arenas in vivo that involve MVBs or exosomes

As pointed out by van Niel et al, "Exosomes are present in the culture supernatant of several cell types of hematopoietic origin [B cells, dendritic cells, mast cells, T cells and platelets] and of non hematopoietic origin [intestinal epithelial cells, tumor cells, Schwann cells and neuronal cells]." [1]; their list also could have included reticulocytes, astrocytes, etc. Although a portion of circulating RNA found in blood, urine and bodily fluids may come from dying cells [53] and microvesicles, at least a significant portion arises from exosomes [54], which implies that exosomes are secreted in vivo, and that one may gain biomedical insights from isolating exosomes from these sources.

A few years ago, "argosomes" were identified as vesicles that transfer morphogenetic signals among cells in developing epithelia. These were shown to arise from endocytic membranes that contain lipid-rich rafts, which are highly reminiscent of (and possibly identical to) exosomes [55].

Last, but not least, the packaging of HIV-1 and other retroviruses into membrane-surrounded virions appears to employ the same cellular process that normally forms multivesicular bodies [56]. This "Trojan exosome" hypothesis [57] has been widely discussed and debated [58], and has recently gained additional support [31,32].

### 4. Exosomal signaling in the central nervous system

Faure et al. have demonstrated that rat and mouse cortical neurons secrete exosomes in culture that have the typical features (size, density and saponin sensitivity) seen in other cell types [51]. Using proteomic methods, they found that neuronal exosomes largely resemble those of non-neural cell types, e.g. expressing Alix, Tsg101, tubulin, 14-3-3 proteins, annexins, clathrin heavy chain, HSC70, GAPDH, etc. [3-10]. In addition, the exosomes contained neuron-specific components. For example, AMPA receptor subunit GluR2/3 was detected within purified neuronal exosomes, but in contrast, NMDA receptor subunit NR1 and PSD-95 (which are primarily found in the PSD fraction of mature synapses in vivo) were not detectable [51]. Neuronal-specific cell adhesion molecule L1 was detected, as was cellular prion protein. The secretion of exosomes (as measured by content of Alix, Tsg101 and GluR2/3) was markedly stimulated by K^+ ^depolarization, which increases levels of intracellular calcium.

Exosomes have also been shown to be secreted by cultured astrocytes [43,59]; because they carry HSP70 which has neuroprotective effects upon neurons, exosomes may contribute to glial-neuronal communication [43,59].

Multivesicular bodies have a generally accepted role in routing proteins for lysosomal degradation, and fusion of MVBs with the plasma membrane is one of the major means by which exosomes are secreted from cells. However, in neurons, a third role for MVBs has been identified [60,61]: Namely, trk-dependent neurotrophins that are taken up from presynaptic terminals stably reside within MVBs during their retrograde transport from synapses, and are then released intracellularly into the cytoplasm upon arrival at the cell body. This process can be conceptualized as the inverse of MVB-mediated exosome secretion, since neurotrophins are taken up from neighboring cells, placed into MVBs, and eventually released into the cytoplasm [60,61]. In both scenarios, however, MVBs participate in a unified system of cell-to-cell signaling.

Finally, MVBs are implicated in the pathogenesis of a number of neurodegenerative diseases. For example, prions are secreted via exosomes in a variety of cell types, and may be a major mechanism of their spread throughout the brain [62]. The beta-amyloid peptide of Alzheimer disease accumulates in MVBs and disrupts the normal function of MVBs to degrade proteins, which may play a role in the pathogenesis of AD [63]. Beta-amyloid peptide is secreted, in part, via exosomes [64]. Mutations in the endosomal ESCRTIII-complex subunit CHMP2B, which affect MVBs and lysosomal degradation, result in frontotemporal dementia [65].

### 5. A model of trans-synaptic exosomal signaling in adult mammalian forebrain

The evidence reviewed so far indicates that secretory exosomes mediate a widespread mode of communication utilized by many cell types, including neurons and astrocytes. Although, to date, most studies of exosomes have been devoted to blood cells, cells involved in cancer and immune function, or cells infected with viruses, I suggest that exosomal signaling may be equally important in the nervous system. Exosomal signaling may potentially operate across a variety of species, and during development as well as in the mature brain. It is not known whether, in general, the secretion and uptake of neuronal exosomes are independent of sites of synaptic transmission; the MVB-dependent trafficking of viruses and neurotrophins seems to occur preferentially and bi-directionally across synapses, which may argue for an association of exosomes with synapses. In one particular case – that of excitatory synapses within the mature adult mammalian forebrain – the evidence for exosomal signaling in vivo is particularly strong, and the biological significance as a mechanism of retrograde signaling at synapses is particularly apparent.

Namely, a large number of diverse observations favor the hypothesis that:

▪**exosomes form at the postsynaptic membrane of excitatory synapses, adjacent to the postsynaptic density, **and

▪**transfer a specific set of synaptic proteins, mRNAs and microRNAs to the presynaptic terminal.**

▪**These cargoes either act locally within the presynaptic terminal or are transported back to presynaptic neuronal cell bodies.**

▪**Exosomal transfer is greatly stimulated by stimuli that elicit LTP and other long-lasting changes associated with learning and memory.**

In this scenario, exosomal transfer of cargoes should comprise one of the means by which retrograde signaling contributes to synaptic plasticity and to trophic maintenance of neuronal circuitry. In the following sections, the evidence for this hypothesis is reviewed in detail.

#### 5a. The postsynaptic membrane expresses endocytic-like lipid raft domains and the dendritic spine contains endosomal sorting structures

Synapses harbor endocytic-like lipid raft domains of the plasma membrane that are located lateral to the postsynaptic density [66,67]. These dendritic lipid rafts are known to be platforms for signal transduction initiated by several classes of neurotrophic factors [66], and are sites of vesicular trafficking of AMPA receptors [68] and new membrane expansion [69] via recycling endosomes. As well, a variety of endocytic structures have been described within dendritic spines, including clearly identifiable multivesicular bodies, as well as other tubular compartments and small vesicles that were identified as potential exosomes [70,71]. While recycling endosomes are not, themselves, sources of exosomal secretion, there is evidence that GluR2 can be diverted to late endosomes/lysosomes in response to NMDA stimulation [72], indicating the existence of a sorting way-station similar to that which occurs within multivesicular bodies. Thus, on the postsynaptic membrane as well as within the dendritic spine, membranous structures exist that may potentially form exosomes.

#### 5b. Synaptic spinules appear to represent exosomes that bud directly into the presynaptic terminal

**Figure 2:**
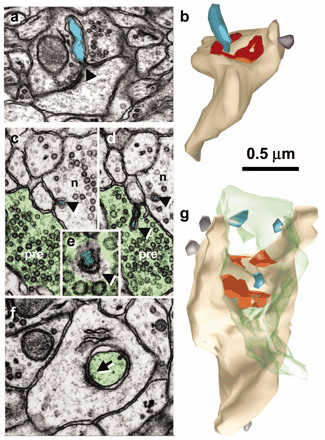
Spinules on mushroom dendritic spines. *a*, Micrograph through the center of a spinule (turquoise) emerging from a perforation (arrowhead) in the postsynaptic density into the presynaptic axon. *b*, Reconstruction of the spine illustrated in *a *with the spinule in turquoise and the PSD surface area in red. *c, d*, Serial sections through the presynaptic axon (pre; green) and a spinule (arrowhead; turquoise) emerging from the edge of a mushroom spine head into the presynaptic axon and another spinule also emerging from the edge of the spine head (arrowhead; lavender) but invaginating a neighboring axon (n). *e*, High magnification of serial section beyond *d *showing a coating along the cytoplasmic surface of the spinule on the side of the invaginated presynaptic axon (arrowhead). *f*, Later sections of the mushroom spine head showing where the presynaptic axon deeply invaginated the spine head in a vesicle-free zone adjacent to a cell-adhesion (arrow) that is adjacent to the postsynaptic density on subsequent serial sections. *g*, Three-dimensional reconstruction of the mushroom spine (beige) with perforated synapse (red) and several small spinules into the presynaptic axon (turquoise spinules) or neighboring axon (lavender spinules). Reproduced from ref. 76 with the permission of the Society for Neuroscience (copyright 2004) and Dr. Harris.

**Figure 3:**
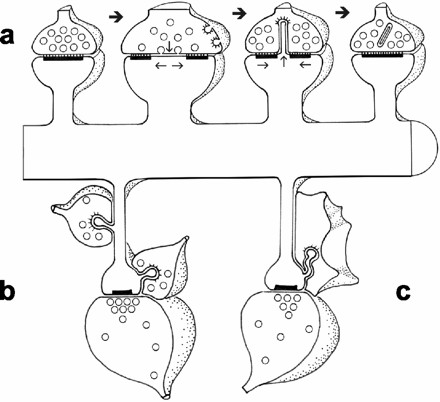
Models of spinule functions. *a*, Process whereby trans-endocytosis of spinules removes excess presynaptic and postsynaptic plasma membrane after substantial activation results in transient perforated or segmented synapses on mushroom spines. This process could also provide retrograde signaling. *b*, Neighboring axons vying for synapses via spinules on the necks and heads of thin spines. *c*, Intercellular signaling between thin spines and perisynaptic glia via spinules. Reproduced from ref. 76 with the permission of the Society of Neuroscience (copyright 2004) and Dr. Harris.

Synaptic spinules are evaginations of the postsynaptic membrane that arise from sites adjacent to postsynaptic densities, that extend into the presynaptic axon. and that appear to be engulfed by the latter into clathrin-coated pits and tubules [73-76] (see fig. 2, 3). Less commonly, spinules arise from axons and growth cones and can also extend into perisynaptic glial cells. Spinules are more conspicuous after eliciting LTP [74-77] and in large mushroom spines [76], suggesting a positive correlation between LTP, spine growth, protein synthesis within the spine head and trans-endocytosis into the presynaptic terminal [76,78]. Investigators have repeatedly suggested that spinules may provide a mechanism for retrograde transfer of cytoplasmic and membrane materials [73,76,79]. The spine apparatus (a specialized membranous structure resembling ER), multivesicular bodies and ribosomes are often found near spinules [73,74], and Tarrant and Routteberg [73,74] have reported that "ribosomal-like" material can be observed within spinule cytoplasm as well, though they have not been reported to contain mitochondria or other organelles. Because of their location, size and activity-dependent regulation, it is likely that synaptic spinules are the morphologic correlate of budding exosomes transferring their cargoes at excitatory synapses.

#### 5c. Neurotropic viruses utilize MVBs and lipid raft domains of the postsynaptic membrane

Many neurotropic viruses that attack the CNS (including polio, rabies, measles, varicella, herpes simplex viruses, etc.) spread preferentially in a trans-synaptic manner, both anterogradely and retrogradely. The Trojan exosome hypothesis was formulated for retroviruses [57], but other viruses may become packaged via exosomal pathways as well; e.g., at least one DNA neurotropic virus, herpes simplex virus type 1, which shows preferential trans-neuronal spread, has been shown to employ multivesicular bodies in its biogenesis [80,81]. In the anterograde direction, virions are packaged within perikarya, are then transported down axons (piggybacking on normal axonal transport mechanisms) and arrive at nerve terminals, where they are released onto neighboring postsynaptic neurons [82]. The preferential targeting of virions to synaptic regions may be facilitated by the fact that the viral proteins and RNAs are transported to nerve endings for assembly [83]; indeed, in sensory neurons, herpesvirus induces the formation of new presynaptic varicosities that serve as assembly and release sites [84]. Conversely, viruses can also be localized to dendritic spines and postsynaptic densities [85] and can spread retrogradely to presynaptic neurons. In fact, under some conditions viral budding has been observed to occur from a site adjacent to the postsynaptic density where they evaginate directly into the presynaptic axon [86] – very reminiscent of synaptic spinules.

#### 5d. Cargo proteins within postsynaptic exosomes

Several proteins that are expressed near the postsynaptic membrane, and that regulate synaptic plasticity, are leading candidates to be carried as cargoes by postsynaptic exosomes:

##### a) CAM kinase II alpha

Subcellular fractionation studies have revealed that a portion of CAM kinase II alpha is associated with dendritic lipid rafts [87,88]. Whereas CAM kinase II has been the subject of extraordinarily intense study because it is critical for postsynaptic mechanisms of plasticity [89], studies have indicated that *presynaptic*CAM kinase II plays a role in synaptic plasticity as well [90-94]. Thus, movement of CAM kinase II protein (or its mRNA) from the postsynaptic to the presynaptic side would be expected to alter synaptic plasticity. In fact, Ninan and Arancio [93] demonstrated that injecting CAMKII alpha protein into presynaptic hippocampal neurons caused potentiation across the synapse when paired with a weak stimulus train. CAMKII holoenzyme at synapses consists solely of alpha subunits organized into a 12-subunit multimer that can form higher-order structures at the synapse [88]. The higher-order multimerization is enhanced by conditions that raise intracellular calcium levels [88], which is noteworthy insofar as Fang et al. proposed that higher-order oligomerization and membrane association are predictive of sorting to exosomes [32]. In carp retina, CAM kinase II has been observed within synaptic spinules [95], though to my knowledge, mammalian spinules have not been examined. Thus, CAM kinase II alpha can be regarded as a leading candidate cargo protein for synaptic exosomes.

##### b) AMPA receptors

These are expressed in the postsynaptic membrane as tetramers, and a portion are associated with lipid rafts [e.g., [87,96]]. These tetramers form larger clusters that vary in size according to stimuli such as conditions that elicit long-term potentiation [96]. Detergent extraction experiments indicate that a significant fraction of the AMPA receptor clusters are found within the lipid rafts [97]. These in vivo considerations suggest that higher-order oligomerization of AMPA receptors can be induced to occur within lipid rafts, and are consistent with the observation that AMPA receptor subunits are detected within purified neuronal exosomes in vitro[51]. (In contrast, NMDA receptors and PSD-95, which in mature synapses in vivo are primarily associated with the postsynaptic density, are not detectable in neuronal exosomes [51].) In fish retina, AMPA receptor subunit GluR2 has been observed within synaptic spinules [98]. Cycling of cell-surface AMPA receptors to and from the cell surface is an important mechanism of regulating synaptic plasticity [89], whereas to my knowledge, no physiologic role for presynaptic AMPA receptors has been described. Thus, packaging AMPA receptors into exosomes may be an additional means by which the postsynaptic (host) neuron rids itself of excess receptors, in addition to the currently known mechanisms (internalization into recycling endosomes and routing to the endosomal/lysosomal pathway for degradation).

##### c) Transcription factors

Engrailed-1 mRNA is transported into dendrites where it is translated locally in response to synaptic activity [99]. As a homeodomain messenger protein, it has been implicated in intercellular movement among cells [100]. In a non-neural cell type, the related protein Engrailed-2 has been found to associate with cholesterol rich endocytic vesicles [48]. As a transcription factor, Engrailed-1 would be expected to alter gene expression within the presynaptic neuron. A variety of other transcription factors are also known to be expressed locally in dendrites, including CREB, NFkappaB, STAT3, NAC1 and Tbr-1, and these also undergo induced mobilization in response to synaptic activity. Although current thinking is that these transcription factors translocate to the cell body, the possibility that they also show movement into exosomes should be examined.

#### 5e. mRNAs as cargoes of postsynaptic exosomes?

If mast cell exosomes do, indeed, harbor mRNAs and microRNAs, as reported by Valadi et al. [3], then it is reasonable to expect that this will prove to be a general feature of exosomes in other cell types. However, as discussed above, this report is not the only reason to suspect that RNA signaling may occur via exosomes, and many pieces of circumstantial evidence are consistent with a role for inter-cellular transfer of mRNAs and microRNAs at synapses:

##### a) A specific population of synaptic mRNAs is transported selectively to dendrites in an activity-dependent manner

This is the case for the candidate exosomal cargoes discussed above, e.g., Engrailed-1, CAM kinase II alpha [101], and AMPA receptor subunits GluR1-3, as well as translation elongation factors 1A and 2 (note that translation initiation factors and elongation factors have been routinely detected within exosomal preparations [3-10]). In fact, polyribosomes (and hence, both mRNAs and newly synthesized proteins) actively move into dendritic spines after LTP eliciting stimuli [102,103]. This stream of transported mRNAs provides a local pool that may potentially become packaged into exosomes.

##### b) Synaptic mRNAs are surprisingly diverse, and are expressed surprisingly close to the postsynaptic membrane

For example, Suzuki et al. prepared postsynaptic density fractions from rat forebrain and identified mRNAs that were enriched in this fraction by gene chip analysis [104]. They found ~1900 different mRNAs, which comprised a number of different functional categories including channels, receptors for neurotransmitters and neuromodulators, integrins and matrix proteins, proteins involved in signaling, scaffold and adaptor proteins and cytoskeletal proteins. Many of these mRNAs were greatly enriched in the PSD fraction relative to total forebrain homogenate (or were undetectable within the total homogenate) [104].

##### c) EIF4E, which binds the 5'-cap of mRNAs during their transport into dendrites, is a good candidate to assist in sorting of mRNAs into exosomes

In subcellular fractionation studies, EIF4E is partitioned to lipid rafts and is localized right next to the PSD. When Asaki et al. [105] characterized the biochemical and EM immunocytochemical distribution of translation initiation and elongation factors within synaptic preparations of mature rat brain, they found that EIF4E, 4E-binding protein, EIF2A, EIF4G, and elongation factor 2 were all preferentially associated with lipid rafts. In their words [105]:

"The eIF4E-immunoreactivity was localized to the postsynaptic sites, especially to the microvesicle-like structures underneath the postsynaptic membrane in the spine, some of which were localized in close proximity to the PSD."

The authors interpreted these results as suggesting that the postsynaptic local translational system takes place, at least partly, immediately beneath the postsynaptic membrane. However, the results also show that the initiation and elongation factors (and presumably their associated mRNAs) are present at or near the proposed sites of nascent exosomes. One can envision why the two processes (protein synthesis and exosomal packaging) might be closely linked: By packaging proteins within exosomes immediately after their synthesis, or by packaging mRNAs to be translated within the recipient neuron, one can ensure that the proteins are "fresh" and functional within the recipient cell and not already involved in stable associations with other proteins.

Besides helping to package mRNAs, translation initiation and elongation factors may also serve as cargo proteins to facilitate protein synthesis within the presynaptic terminal. Although growing axons and growth cones have active protein translation machineries, protein synthesis is relatively sparse in mature presynaptic terminals [106]. Thus, even a relatively small transfer of regulatory proteins and mRNAs to the presynaptic side might have a significant contribution to overall protein synthesis in this location.

Another candidate RNA-binding cargo protein is CPEB1, a protein that binds to CPE elements present in the 3'-UTR of certain mRNAs [107]. CPEB1 is a key regulator of mRNA transport in neurons: For example, it is a component of RNA transport particles that contain staufen and FMRP [108]. CPEB1 stimulates transport of CAM kinase II alpha mRNA to dendrites in an activity-dependent manner [109]; and it works in conjunction with EIF4E and other proteins to regulate the polyadenylation and translation of the mRNAs that it binds, many of which encode mRNAs that are transported to dendrites and that encode synaptic proteins [110]. To my knowledge, there is no direct evidence that CPEB1 associates with lipid rafts at synapses. However, Cao et al. [111] showed that CPEB1, as well as a variety of translational components including EIF4E and CPSF, show an association with oocyte membranes through binding to the C-terminal tails of amyloid precursor proteins APP, APLP1 and APLP2. This is noteworthy since amyloid precursor family proteins are enriched at synaptic sites, can be routed to lipid rafts [112], and have been identified within endosomal-lysosomal compartments as well. Furthermore, the association with APP family proteins promotes CPEB1-dependent polyadenylation and translation [111].

##### d) A compartmentalized population of microRNAs is also expressed in synaptic fractions

Hundreds of microRNAs are expressed within the mature forebrain, and at least some regulate the translation of specific target mRNAs within dendritic spines [[113], reviewed in [114]]. My group has recently characterized the relative abundance of mature microRNAs in microarray and real time RT-PCR studies of synaptic fractions (i.e., synaptoneurosomes) isolated from adult mouse forebrain. Most of the microRNAs that are expressed in the forebrain homogenate are detectable in synaptoneurosomes as well; some microRNAs are much (up to ~8-fold) less abundant in the synaptic fraction, but most have roughly equal abundance, and about 10% of the microRNAs are significantly enriched in the synaptic fraction (Lugli et al., ms. in preparation). Thus, the population of microRNAs is abundant, diverse and has a characteristic composition near mature synapses. It is not clear how microRNAs would be sorted into exosomes, but most microRNAs are found in association with actively translating mRNAs on polyribosomes [115]. If synaptic mRNAs or microRNA-binding proteins (such as FMRP or EIF2c) are sorted into exosomes, they could conceivably carry the microRNAs with them.

### 6. Testing the model

Does exosomal trans-synaptic signaling occur within the mammalian CNS? If so, do exosomes transfer proteins, mRNAs or microRNAs from postsynaptic dendritic spines to presynaptic terminals? Several lines of experiments are proposed to test these hypotheses:

#### a) Tests of neuronal exosomes in vitro

Do neuronal exosomes [51] express any of the predicted synaptic cargo proteins (e.g., CAM kinase II alpha, EIF4E or CPEB1)? Are mRNAs detected? If so, are they enriched in synaptic mRNAs and/or in mRNAs that bear CPE elements? Will depolarization of the neurons increase the abundance or alter the types of secreted RNAs? Are microRNAs detected within exosomes as well? If so, can one identify related proteins such as dicer, FMRP or EIF2c [116]? When neuronal exosomes are co-cultured with naïve recipient neurons, can one demonstrate uptake of RNAs into the recipient cells and show that the exosomes produce appropriate effects, namely translation of transferred mRNAs and silencing of certain endogenous mRNAs due to transferred microRNAs?

#### b) Tests of exosomal transfer in vivo

If CAM kinase II alpha is indeed a cargo for exosomes, can CAM kinase II alpha protein be detected in vivo in non-forebrain neurons that project to the forebrain, e.g., in thalamic projection neurons that are not known to synthesize this protein? If so, then either the CAM kinase II alpha protein (or its mRNA) must have been transferred to the thalamic neurons.

Another test is to characterize synaptic spinules using EM immunocytochemistry, to ask if they harbor exosomal marker proteins (Alix and Tsg101), as well as the putative candidate protein cargoes and mRNAs (using tagged oligo dT probes).

Perhaps most importantly, one should be able to express a tagged version of Alix protein selectively in postnatal forebrain neurons of transgenic mice, by using constructs that are under the control of the CAMKII alpha promoter. (Because overproduction of Alix can lead to cell death via its interactions with ALG-2, a mutated form of Alix lacking the ALG-2 interaction domain should be employed [117]. As a positive control, one needs to verify that exosome secretion occurs normally in cortical neuronal cultures prepared from the transgenic mice, and that one can detect the tagged, mutated Alix protein within exosomes secreted in these cultures.) If tagged Alix protein is detected not only in forebrain neurons, but also within their presynaptic partners in brain regions that lack CAMKII expression, then one can conclude that Alix is being transferred intercellularly, most likely within exosomes. As a negative control, expressing a tagged irrelevant protein thought NOT to be present in exosomes (e.g., PSD-95 [51]) should show no intercellular transfer. If exosomal signaling is verified, then one can tag candidate synaptic proteins and their mRNAs (chosen from among those that are expressed in preparations of neuronal exosomes) and test their intercellular transfer using a similar strategy.

## Conclusion

Ten years ago, the existence of the class of microRNAs and other tiny RNAs was not merely unknown, but entirely unpredicted and unsuspected. Similarly, I believe that exosomal secretion has remained "below the radar" of most neurobiologists because there does not appear to be any *need*for yet another mechanism of cell-cell signaling. Just as the tiny RNAs have reared their heads in too many arenas to be ignored, so have exosomal-like vesicles appeared in many guises including argosomes found in developing epithelia, circulating fetal RNA found in the maternal blood, tissue antigens processed by antigen-presenting cells, and synaptic spinules.

My contribution in this review has been to draw attention to the likelihood that exosomal signaling is a fundamental mode of communication within the nervous system, and that not only proteins but also mRNAs and microRNAs may be transferred, thus supplementing the known mechanisms of anterograde and retrograde signaling across synapses. In particular, I propose that lipid raft regions of the postsynaptic plasma membrane are sites of exosomal packaging and intercellular transfer of synaptic signaling molecules that play key roles in regulating synaptic plasticity and long-term trophic effects on neural circuitry. Exosomes are likely to be important even if further tests reveal that they only carry proteins (and not RNAs). However, it will be interesting to see whether, in ten years, *inter*-cellular RNA signaling achieves the same level of respectability and interest that *intra*-cellular RNA signaling has today.

## Review 1

Dr. Etienne Joly, Equipe de Neuro-Immuno-Génétique Moléculaire, CNRS, France

In this manuscript, Neil R. Smalheiser proposes that inter-neuronal communication in the nervous system could involve the inter-cellular transfer of macromolecules (proteins and RNAs) via ferrying by exosomes. I am probably not the most impartial judge for these ideas because I have also been convinced for quite a while that the phenomenon of using macromolecules as inter-cellular messengers does exist, and probably not just between the cells of the central nervous system, but on a very large and frequent scale.

In my eyes, the main strength of this manuscript lies with the large number of observations from a vast number of published papers (139 in total) from very diverse areas of biology that Neil Smalheiser has managed to collect to strengthen his hypothesis, many of which had escaped my attention.

This being said, I must admit that I am slightly surprised that the author should be so convinced that the traffic of macromolecules between cells could only be conveyed by exosome-like vesicles, and has not mentioned nanotubes or gap junctions as potential routes of exchange for RNAs & protein.

Another initial criticism I had made regarding the manuscript was that it did not make for very easy reading, but I have found the current version to be much improved.

### Author's response

I have altered the focus of the review – instead of covering the topic of intercellular RNA signaling in general, and then discussing a specific example in the CNS, I have now gone straight to the topic of exosomes, and approached the model from that perspective. This makes the paper shorter and less meandering, and I have rearranged and rewritten much of it. I have also added a paragraph that mentions a variety of possible mechanisms besides exosomes that can potentially support intercellular RNA signaling.

## Review 2

Dr. Gaspar Jekely, Max Planck Institute for Developmental Biology, Germany

This paper presents the interesting hypothesis that secreted exosomes can function in intercellular signaling in the nervous system. The author provides an extensive review of the literature of exosomes and exosome-mediated signaling and formulates the hypothesis that cargo proteins and RNAs of exosomes can have important roles in neuron to neuron signaling. Importantly, feasible experimental approaches to test the hypothesis and specific candidate proteins that possibly mediate intercellular signaling are also suggested. This hypothesis will surely increase awareness of the multiple roles of exosomes in cell and neurobiology and will potentially stimulate important experimental work.

A few additions and clarifications would help in presenting the model and its predictions. Since the generation and secretion of exosomes is a multi-step process involving several regulated topology-breaking membrane rearrangements, the paper would greatly benefit from a figure and an explicit listing of all the necessary steps that would have to occur if exosome signaling is to work. These steps include at least the sorting of lipids, proteins and RNAs at the plasma membrane and subsequent inward budding to generate an endosome, the sorting steps and inward budding of this vesicle to generate MVBs, the regulated (activity-dependent) fusion of MVBs with the plasma membrane to secrete exosomes, the fusion of exosomes with the target cell and the signaling step within the target cell.

### Author's response

I have now added a figure on exosomes (and two on synaptic spinules). The revised version also discusses the Faure et al. paper in more detail, and changes the title to reflect the scope better.

## Review 3

Juergen Brosius, Institute of Experimental Pathology/Molecular Neurobiology, University of Muenster, Germany

The idea of intercellular RNA has been around for some time. While there is more and more evidence in plants, e.g., see: Dunoyer P, Himber C, Ruiz-Ferrer V, Alioua A, Voinnet O. (2007) Intra- and intercellular RNA interference in Arabidopsis thaliana requires components of the microRNA and heterochromatic silencing pathways. Nat Genet. 39:848–56. Epub 2007 Jun 10, the situation is still highly speculative in mammals. Nevertheless, one continues to finds hints (as outlined in the manuscript) that intercellular RNA signaling could also play a role in mammals, especially in the nervous system. About two decades ago, Steven Benner has made such an argument based on reports of cytotoxicity of RNAses by extracellular action: Benner SA. (1988) Extracellular 'communicator RNA'. FEBS Lett. 233:225–8. This should be mentioned.

Even today, unequivocal experimental evidence will be hard to come by (clean sub-cellular fractions, avoidance of "extracellular" RNA by lysed cells, etc.). The author has done an extraordinary job to describe (and underscore with existing information) some scenarios that could happen if there was intercellular RNA signaling in mammals. Predictions of the potential harvest will hopefully encourage future efforts despite the expected experimental pitfalls. This alone would justify publication of this piece.

The danger of collections of such "Texas sharpshooter fallacies"  lies in the high probability that at least one component has to be (re)moved, leading to the collapse of sections or the entire "house of cards".

### Author's response

I have added a paragraph that discusses Benner's paper, among other possible mechanisms of inter-cellular RNA signaling that were not discussed in the original manuscript (e.g. gap junctions). I have also rewritten the paper to acknowledge, yet avoid as much as possible, the "house of cards" issue. That is, even if the paper of Valadi et al. (claiming that mast cell exosomes harbor mRNAs and microRNAs) cannot be replicated, I try to explain how and why my model still has a leg to stand on.

## Review 4

Dr. Eugene Koonin, National Center for Biotechnology Information, NIH, USA

This is, decidedly, a very educational and provocative paper. That RNA might be a major agent of cell-to-cell communication in mammals is an exciting possibility, and I agree with Smalheiser that there is a chance that this is a major signaling pathway that goes "under the radar" much like small regulatory RNAs did just a few years ago. Only a chance but that is already a good reason to spell it out and highlight the possibility. This being said, I find many arguments in the paper to be somewhat less then persuasive or, perhaps, not argued as closely as possible.

1. I am not quite compelled to believe that there are systems of intercellular RNA signaling in invertebrates beyond pathogen response. Such systems certainly do exist in plants but, with the extensive cell-to-cell communication through the plasmodesamata, this is a different story. Perhaps, it is possible to elaborate on this point.

2. I am not sure about the relevance of the description of the secretion of retroviruses. Then, again, if retroviruses are relevant, why not all kinds of enveloped viruses? These are, indeed, brought into the fold later in the paper. If this is all about the Trojan exosome hypothesis, i.e, viruses exploiting normal mechanisms of RNA secretion, then, this has to better explained and critically assessed.

3. The argument very heavily relies on the paper by Valadi et al. (Ref. 3). All other reports on cell-to-cell communication in mammals are either tentative, as noted by Smalheiser, or tangentially relevant (like the liposome experiments). On a more positive note, the evidence of exosomal origin of much of the RNA found in blood etc (reviewed in ref. 54) is suggestive.

4. I find it rather strange that the discussion of the proteins that might be packaged into synaptic exosomes is much more detailed than the corresponding discussion of mRNAs and miRNAs. The paper is generally about RNA-based cell-to-cell signaling not about accompanying proteins. I would think there should be some more detail on synaptic RNAs and less text on proteins.

### Author's response

Most of these issues were dealt with by refocusing the paper on exosomes (and considering both protein and RNA cargoes), since this is at the heart of the specific model I am proposing (retrograde movement of exosomes at synapses). I do not believe that viral packaging into exosomes necessarily implies that RNAs are packaged into exosomes in the same way, and I don't think that implication comes across in the revised version.

## Competing interests

The author declares that he has no competing interests.
